# Is standard total knee arthroplasty with lateral femoral overhanging a cause of anterior knee pain? A randomized controlled trial

**DOI:** 10.1051/sicotj/2022003

**Published:** 2022-02-22

**Authors:** Boonchana Pongcharoen, Narong Tantarak, Waroot Pholsawatchai

**Affiliations:** 1 Department of Orthopaedic Surgery, Thammasat University 95 Paholyothin Road KlongLuang 12120 Thailand; 2 Chulabhorn International Collage of Medicine, Thammasat University 99 Moo 18 Paholyothin Road Pathumthani 12120 Thailand

**Keywords:** Anterior knee pain, Gender-specific TKA, Unisex TKA, Patellar tracking, Femoral component overhanging

## Abstract

*Introduction*: Anterior knee pain (AKP) may persist after total knee arthroplasty (TKA), even if well aligned and stable, and is reported in up to 30% of patients, leading to patient dissatisfaction. The gender-specific knee prostheses have been designed to reduce femoral component overhanging in females and improve patient satisfaction. The purpose of this study was to determine AKP between gender-specific knee prosthesis and unisex knee prosthesis following minimally invasive surgery (MIS) TKA with patellar resurfacing. *Methods*: This study was a randomized trial comparing a gender-specific vs. unisex knee prosthesis in females with knee osteoarthritis. Follow-up occurred at 6 weeks, 3 months, 6 months, 1 year, and 2 years. Pre- and postoperative AKP were measured at each follow-up. Intraoperative lateral overhanging of the femoral component and patellar tracking were also measured and compared between the two groups. *Results*: Sixty females were recruited; 30 underwent gender-specific knee prosthesis (Gp1) and 30 underwent unisex knee prosthesis (Gp2). No patients were lost to follow-up. The incidence rates of AKP and visual analog scale AKP pain scores at 2 years were 7 vs. 7% (*p* = 1.00) and 0.95 ± 0.31 (0–1) points vs. 1.10 ± 0.28 (0–1) points (*p* = 0.68) for gender and unisex prostheses, respectively. Patellar tilt and patellar shift were similar between the two groups. Patellar tilt and patellar shift were 2.56° ± 2.03 (0–8) vs. 2.67° ± 2.35 (0–9) (*p* = 0.46) and 1.25 ± 1.09 (0–3.2) mm vs. 1.15 ± 0.97 (0–2.9) mm (*p* = 0.34) for Gp1 and Gp2, respectively. Mean lateral femoral overhanging was 0.23 ± 0.63 mm (range: 1–2 mm, Gp1) vs. 1.57 ± 1.36 mm (range: 1–3 mm, Gp2) (*p ≤* 0.001). *Conclusion*: Both types of prostheses had similar incidence rates of AKP, VAS scores for AKP. Lateral femoral overhanging of ≤ 3 mm was not the cause of AKP.

## Introduction

Total knee arthroplasty (TKA) has shown good outcomes and survivorship [1–5]. However, postoperative anterior knee pain (AKP) is a common problem after TKA and affects patient satisfaction [6–9]. The reported incidence of AKP after TKA is 3–30% [6–9]. The causes of AKP after TKA were divided as follows: surgical technique factors such as instability, malalignment of the prosthesis, patellar maltracking, and overhang of implant [10–14]; implant factors such as posterior stabilized TKA with high intercondylar box, too large and thick of anterior condyle, shallow trochlea groove [3–5]; and patient factors such as female, obesity, and high Q-angle [15–21]. Females tended to develop AKP even when they had good TKA, because females have greater anteroposterior (AP)/mediolateral (ML) ratios and smaller anterior condyles. Therefore, overhang of a femoral prosthesis and overstuffing of the patella may be the cause of AKP [15–17]. Consequently, gender-specific knee prostheses have been designed for women to reduce the AP:ML ratio of overhang of the femoral component, increase the valgus angle of the trochlea groove, restore the Q-angle, and reduce the thickness of the anterior part of prosthesis to prevent overstuffing of the patella. However, previous studies of gender-specific knee prostheses have shown similar clinical outcomes as unisex knee prosthesis. The only apparent benefit of a gender-specific knee prosthesis was a reduction in the overhanging of the prosthesis [17–22]. There has been no study comparing the AKP of unisex knee prosthesis with overhanging of the femoral component and gender-specific knee prostheses following minimally invasive surgery (MIS) TKA. The purpose of this study was to determine whether AKP and patellar tracking differ between gender-specific knee prosthesis and unisex knee prostheses following MIS TKA with patellar resurfacing.

## Materials and methods

We conducted a prospective randomized trial study from January 2013 to July 2016 at Thammasat University hospital, Pathumthani, Thailand. The Human Research Ethics Committee of the Faculty of Medicine, Thammasat University, approved the study (Reg. no: MTU-EC-OT-1-005/55). The clinical trial number was NCT05045651.

**Figure 1:**
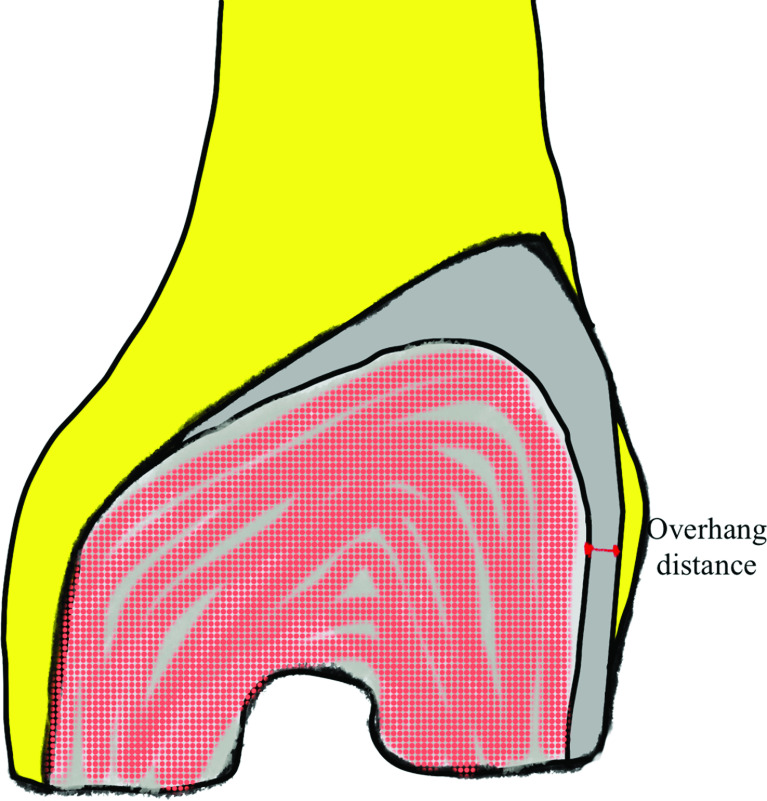
Lateral overhanging of the femoral component was measured intraoperatively. The maximum distance between the lateral edge of the femoral component and lateral bone cut edge was measured twice by the surgeon and assistant.

**Figure 2:**
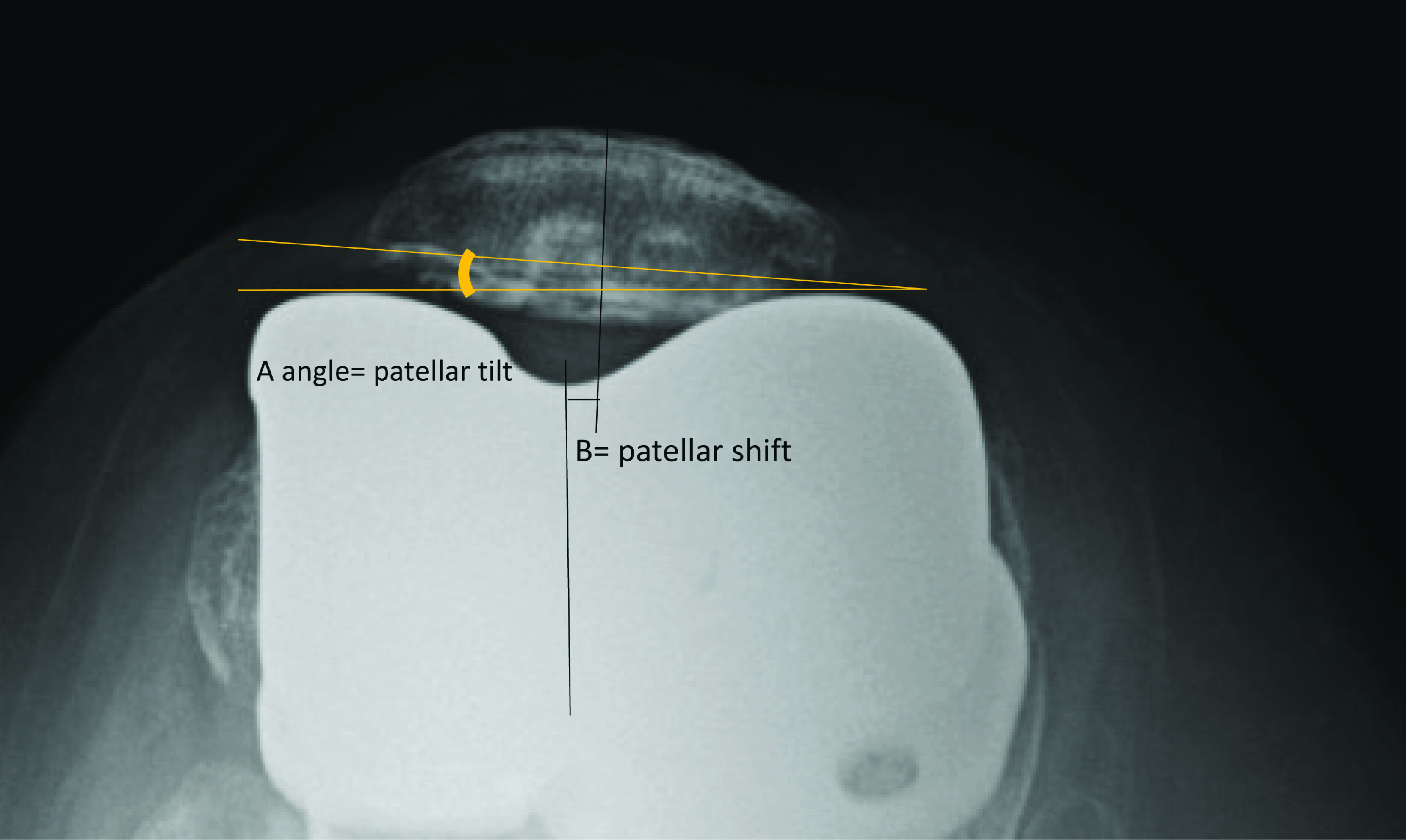
The A angle is patellar tilt. The B distance is patellar shift. It is the distance between center of trochlea of prosthesis and center of patella is patellar shift.

We included female patients with osteoarthritis (OA) of the knee who were older than 50 years of age, with a range of movement (ROM) > 90°, a varus deformity < 25°, genu recurvatum < 20°, and flexion contracture < 20°. Exclusion criteria were spontaneous osteonecrosis of knee (SPONK), inflammatory joint arthritis, and post-traumatic arthritis. Patients were randomized into two groups (Gp) according to a computer-generated randomization list with block randomization, and the block size was two: (i) Gp1 – gender-specific knee prosthesis (posterior stabilized Gender Solution^®^ NexGen^®^ LPS Hi-Flex), and (ii) Gp2 – unisex prosthesis (posterior stabilized NexGen^®^ LPS Hi-Flex), both produced by Zimmer, Inc, Warsaw, IN, USA. All procedures, which included patellar resurfacing in all cases, were performed by a single surgeon (BP). The maximum lateral femoral overhanging of metal on the lateral bone cut edge was measured and recorded intraoperatively by BP and NT (Figure 1). Interobserver reliability also was calculated. Patients were followed up at 6 weeks, 3 and 6 months, and 1 and 2 years. At each follow-up, the patients were asked about AKP during low chair-rising activities and stair climbing, and the degree of AKP pain was recorded using a visual analog scale (VAS) from 0 (no pain) to 10 (maximal pain). The Knee Society score (KSS) and range of movement (ROM) were also recorded. At each follow-up, knee X-rays were performed, including anteroposterior (AP) lateral standing and patellar skyline views, to record prosthesis alignment and patellar tilt and shift using Gomes’s technique (Figure 2) [23]. Patient data, including age, gender, site, body mass index (BMI), preoperative ROM, flexion contracture, genu recurvatum, and preoperative knee score, were recorded on a standard case record form. The degree of varus deformity was measured from the standing AP knee X-ray (Table 1).

**Table 1:** Baseline patient characteristics.

Variables	Gp1: Gender-specific knee prosthesis (*n* = 30 knees)	Gp2: unisex knee prosthesis (*n* = 30 knees)	*p*-value
Age (years)*	68.85 ± 6.53 (56–77)	66.73 ± 6.24 (55–78)	0.64
Site (left/right)	26/4	27/4	0.73
BMI (kg/m^2^)*	26.16 ± 4.02 (20–41.62)	25.92 ± 3.74 (21.40–42.22)	0.51
Varus deformity(°)*	8.4 ± 3.02 (0–20)	8.2 ± 3.24 (0–20)	0.43
Flexion contracture (°)*	12 ± 3.67 (5–30)	10 ± 3.67 (5–25)	0.35
Genu recurvatum (°)*	0.41 ± 2.32 (5–15)	0.44 ± 2.46 (5–20)	0.81
KSS (point)*	38 ± 2.45 (28–52)	39.77 ± 2.32 (28–52)	0.23
ROM (°)*	120 ± 12.21 (90–120)	122 ± 12.87 (90–120)	0.73
Incidence of AKP (%)	83.33% (25/30)	80% (24/30)	0.76
VAS for AKP (point)*	2.13 ± 1.90 (0–6)	2.21 ± 2.01 (0–8)	0.35
Patellar tilt (°)*	2.65 ± 2.73 (0–8)	2.78 ± 2.81 (0–8)	0.31
Patellar shift (mm)*	1.86 ± 1.47 (0–6)	1.74 ± 1.51 (0–7)	0.36

KSS: knee society score; ROM: range of motion; BMI: body mass index; AKP: anterior knee pain; VAS: visual analog scale.

* Value are expressed as a mean ± *SD*, with ranges in parentheses.

## Statistically analysis

The sample size was calculated using the minimally clinical important different of VAS for AKP, which was 1.0 point [24], and standard deviation was 1.3. Therefore, 29 patients/arm was 90% power at 5% significance. Preoperative AKP had normal distribution according to the Kolmogorov–Smirnov test (*p* = 0.20) and Shapiro–Wilk test (*p* = 0.76). Continuous data (AKP VAS, patellar tilt, patellar shift, age, BMI, KSS, ROM, posterior slope, femoral component alignment, tibial component alignment, and posterior slope) between the two groups were compared using Student’s *t*-test. The incidence of AKP and knee side were compared using the chi-square test. All analyses were two sided, and a *p*-value of < 0.05 was statistically significant. SPSS version 24 (IBM) was used for analysis of all data.

## Surgical technique and postoperative care

One of us (BP) performed all procedures. An anteromedial skin incision was made from 2 cm above the upper patella pole to the medial aspect of the tibial tubercle. The mini mid-vastus approach was performed in all cases to improve patellar tracking [7]. The distal femur was cut first with 5° of the valgus. The femur size was matched with the size of the prosthesis using the anterior reference and then cut with external rotation at 3°. The proximal tibia was then cut 1–2 mm below the deepest part of bone defect at the medial tibial plateau. The intercondylar notch of the femoral component was performed in the center or with lateralization of the femoral component. The femoral component did not allow medialization of the component. The patella was resurfaced in this step. The trial femoral, tibial, and patella components, and appropriate polyethylene thickness were applied and then the knee was checked for stability, alignment, and patellar tracking. All components were fixed to the bone with bone cement. The distance in millimeter of lateral overhanging of the femoral component was measured by BP and NT (Figure 1). The operative time was recorded.

All patients were encouraged to perform ankle pump and active assistive knee exercises as soon as possible after the TKA and were encouraged to walk with full weight bearing on Days 1 and 2 (the usual day of discharge). All TKAs were performed with the same MIS instrumentation and received the same postoperative pain medications.

## Results

**Figure 3:**
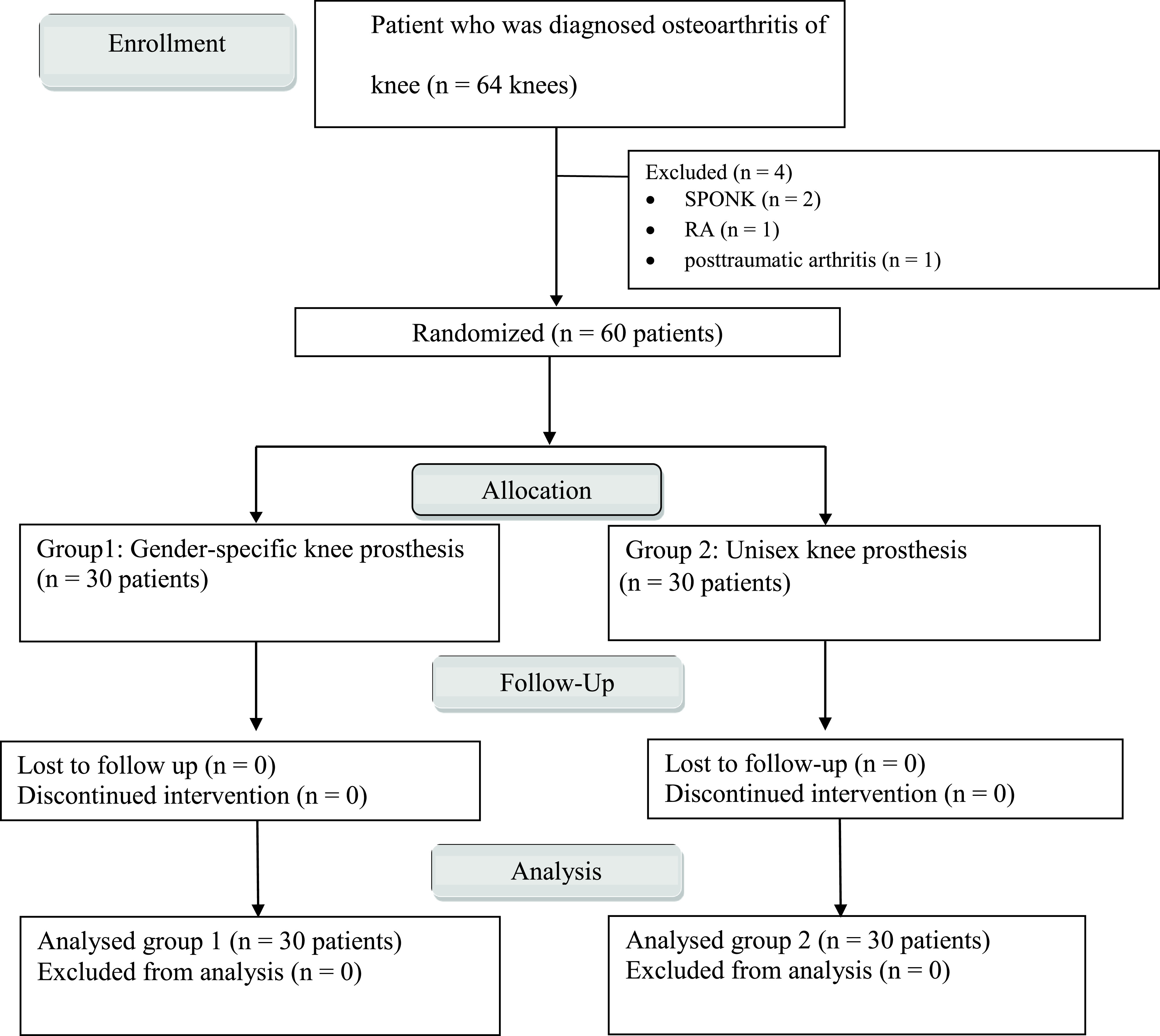
Flow chart protocol of this study (RA: rheumatoid arthritis, SPONK: spontaneous osteonecrosis of knee).

Of the 64 screened women with knee OA, three were excluded, two with SPONK, one with inflammatory joint, and one with posttraumatic arthritis (trial profile, Figure 3). The baseline patient characteristics were similar between the two groups (Table 1).

Both groups (Table 2) had the same incidence rates of AKP and VAS for AKP at all time points. At two years, AKP rates were 6.67% in both groups (*p* = 1.00), and the VAS for AKP were 0.95 ± 0.31 (0–1) (Gp1) vs. 1.10 ± 0.28 (0–1) (Gp2, *p* = 0.68) (Table 2). Patellar tilt and patellar shift did not differ between the two groups at all time points. Patellar tilt and patellar shift at 2 years were 2.56° ± 2.03 (0–8) vs. 2.67° ± 2.35 (0–9) (*p* = 0.46) and 1.25 ± 1.09 (0–3.2) mm vs. 1.15 ± 0.97 (0–2.9) mm (*p* = 0.34) for Gp1 and Gp2, respectively (Table 3). The proportions of patients with lateral overhanging were 66.67% (20/30) vs. 13.33% (4/30) for Gp1 and Gp2, respectively (*p ≤* 0.001). The mean lateral overhanging was 0.23 ± 0.63 mm (range: 1–2 mm, Gp1) vs. 1.57 ± 1.36 mm (range: 1–3 mm, Gp2) (*p ≤* 0.001). Interobserver reliability using Pearson’s correlation was 0.932. The KSS at 6 months, 1 year, and 2 years; ROM at 2 years; operative time; knee alignment; component alignment; and posterior slope were not significantly different between the two groups (Table 4). No patients experienced complications such as patellar clunk syndrome, patellar crepitation, infection, or fracture.

**Table 2:** Incidence of AKP and VAS for AKP.

Variables	Incidence of AKP (%)		*p*-value	VAS for AKP (point)*		*p*-value
	Gp1 (*n* = 30)	Gp2 (*n* = 30)		Gp1 (*n* = 30)	Gp2 (*n* = 30)	
At 6 weeks	33.33% (10/30)	36.67% (11/30)	0.85	4.85 ± 1.71 (0–5)	5.13 ± 1.3 (0–5)	0.49
At 3 months	16.67% (5/30)	23.33% (6/30)	0.78	2.23.0 ± 0.91 (0–4)	2.63 ± 0.90 (0–4)	0.34
At 6 months	16.67% (5/30)	16.67% (5/30)	1.00	2.33 ± 0.82 (0–3)	2.43 ± 0.87 (0–3)	0.74
At 1 year	10% (3/30)	10% (3/30)	1.00	1.24 ± 0.36 (0–1)	1.25 ± 0.39 (0–1)	0.91
At 2 years	6.67% (2/30)	6.67% (2/30)	1.00	0.95 ± 0.31 (0–1)	1.10 ± 0.28 (0–1)	0.68

AKP = anterior knee pain; VAS = visual analog scale.

* Values are expressed as mean ± *SD*, with ranges in parentheses.

**Table 3:** Patellar tilt and patellar shift.

Variables	Patellar tilt (°)*		*p*-value	Patellar shift (mm)*		*p*-value
	Gp1 (*n* = 30)	Gp2 (*n* = 30)		Gp1 (*n* = 30)	Gp2 (*n* = 30)	
At 6 weeks	3.42 ± 2.71 (0–10)	3.21 ± 2.45 (0–10)	0.32	1.24 ± 1.05 (0–3.0)	1.18 ± 0.98 (0–2.7)	0.27
At 3 months	2.66 ± 2.35 (0–8)	2.69 ± 2.41 (0–9)	0.81	1.31 ± 1.09 (0–3.1)	1.26 ± 0.95 (0–3.0)	0.11
At 6 months	2.56 ± 2.03 (0–8)	2.67 ± 2.35 (0–10)	0.49	1.35 ± 1.09 (0–3.2)	1.24 ± 1.02 (0–3.0)	0.47
At 1 year	2.71 ± 2.34 (0–8)	2.82 ± 2.55 (0–9)	0.52	1.26 ± 1.09 (0–3.2)	1.11 ± 1.03 (0–2.7)	0.56
At 2 years	2.56 ± 2.03 (0–8)	2.67 ± 2.35 (0–9)	0.46	1.25 ± 1.09 (0–3.2)	1.15 ± 0.97 (0–2.9)	0.34

* Values are expressed as mean ± *SD*, with ranges in parentheses.

**Table 4:** Secondary outcomes.

Variables	Gp1: Gender-specific knee prosthesis (*n* = 30 knees)	Gp2: unisex knee prosthesis (*n* = 30 knees)	*p*-value
KSS (°)* at 6 months	81.54 ± 14.96 (32–98)	82.02 ± 12.24 (53–99)	0.57
KSS (°)* at 1 year	93.15 ± 8.13 (70–100)	93.78 ± 7.65 (75–100)	0.65
KSS (°)* at 2 years	98.5 ± 2.0 (95–100)	98.7 ± 2.3 (95–100)	0.86
ROM (°)* at 2 years	126 ± 12.32 (90–145)	124 ± 14.02 (90–140)	0.52
Operative time (min)*	90.56 ± 12.46 (55–115)	91.68 ± 13.56 (55–115)	0.43
Knee alignment (°)*	Valgus 5.17 ± 1.32 (2–9)	Valgus 4.93 ± 1.40 (2–9)	0.34
Femoral component alignment (°)*	Valgus 4.08 ± 1.42 (3–6)	Valgus 4.01 ± 1.38 (3–6)	0.82
Tibial component alignment (°)*	Valgus 0.85 ± 1.13 (varus 1 – valgus 3)	Valgus 1.15 ± 1.09 (varus 1– valgus 3)	0.46
Posterior slope (°)*	6.1 ± 1.91 (3–10)	6.4 ± 1.89 (3–10)	0.62

KSS: knee society score; ROM: range of motion.

* Value are expressed as mean ± *SD*, with ranges in parentheses.

## Discussion

Our study revealed that gender-specific knee prosthesis had the same rates of AKP and VAS for AKP with unisex knee prosthesis. A lateral overhanging of the femoral component of ≤ 3 mm was not the cause of AKP. The design of the female-adapted TKA, which aims to reduce the AP:ML ratio of the femoral component, has helped surgeons prevent overhanging of the prosthesis. However, unisex knee prostheses with lateral femoral overhanging ≤ 3 mm had similar rates of AKP and VAS for AKP. Moreover, patellar tracking was also the same for both groups.

Previous studies reported similar results as our study [17–22, 25]; i.e., gender-specific knee prostheses had essentially the same clinical outcomes as unisex prostheses, including good clinical outcomes and survival. However, unlike us, no study has reported on the comparison of both AKP and patellar tracking. In our study, we did not find an advantage of using the gender-adapted prosthesis on the basis of patellar tracking, but it may be useful for reducing femoral overhanging and may reduce the incidence of recut femur.

Our study also found that TKA resulted in a lateral overhanging with a mean of 1.63 mm, ranging from 1 to 3 mm without increasing AKP. Indeed, a small lateral overhanging may improve patellar tracking by reducing the Q-angle without impinging on the soft tissue of the knee to cause pain later on. However, no studies have examined in detail the effects of different degrees of lateral overhanging in TKA, which requires further studies.

Our study had several limitations. First, we did not record the patellofemoral knee score, but this is unlikely to have been different between the two groups, given the similar VAS scores and low incidence of AKP. Second, TKA component alignment was not measured using a knee CT scan, which is more sensitive than conventional X-rays.

In conclusion, the gender-specific knee prosthesis and unisex knee prosthesis had similar AKP and patellar tracking in female patients. The gender-specific knee prosthesis did achieve lower lateral overhanging, but a lateral overhanging ≤ 3 mm did not cause AKP or knee pain.

## Conflicts of interest

The authors declare that they have no conflicts of interest.

## Funding

This research did not receive any specific funding.

## Ethical approval

The Human Research Ethics Committee of the Faculty of Medicine, Thammasat University, approved the study (Reg. no: MTU-EC-OT-1-005/55). The clinical trial number was NCT05045651.

## Informed consent

All patients in this study signed the informed consent statement and agreed to joint this study.

## Author contributions

Boonchana Pongcharoen: Conceptualization of the study, writing- original draft, review and editing.

Narong Tantarak: data collection and statistical analysis.

Waroot Pholsawatchai: data collection and writing-original draft, review and editing.
